# Building Awareness and Engagement: Launching Eckerd College’s Age-Friendly University Status

**DOI:** 10.1093/geroni/igaf122.1932

**Published:** 2025-12-31

**Authors:** Tamar Shovali, Alexis Ramsey-Tobienne, Kat Robinson

**Affiliations:** Eckerd College, St. Petersburg, Florida, United States; Eckerd College, St. Petersburg, Florida, United States; Eckerd College, St. Petersburg, Florida, United States

## Abstract

Eckerd College, a small, private liberal arts college in St. Petersburg, FL, earned the Age-Friendly University (AFU) designation in 2019. Guided by the evidence-based Age Inclusivity Domains of Higher Education (AIDHE) model, we adopted a multifaceted approach to launch our AFU status and foster campus-wide awareness and engagement across the seven domains. The AIDHE model outlines that campus-wide strategies for this launch should focus on raising awareness, providing information and education, and assessing campus constituents. Our first goal was to raise awareness of age diversity, friendliness, and inclusivity on campus. A multi-tiered approach began by forming a coalition with The Inspired Network center for teaching, learning, and faculty development and engaging in conversations with administration and key staff from various domains, including advancement, OLLI, the Academy of Senior Professionals, admissions, and human resources. This was followed by an AFU Lunch and Learn for faculty, staff, and community members to raise broader awareness about our designation, highlight the value of working in an age-diverse environment, and assess attendees’ specific resource needs to inform future practices. To broaden student awareness, a Careers in Aging Month event was planned to provide information about AFUs and opportunities in the field of aging. Subsequently, an assessment of non-traditional student experiences at the college led to the development of a student club designed for this population. Our overarching strategy, other Age-Friendly initiatives aiding in this launch, and specific resource needs will be discussed.

